# The N-terminus of survivin is a mitochondrial-targeting sequence and Src regulator

**DOI:** 10.1242/jcs.183277

**Published:** 2016-07-15

**Authors:** Lucia Dunajová, Emily Cash, Robert Markus, Sophie Rochette, Amelia R. Townley, Sally P. Wheatley

**Affiliations:** School of Life Sciences, University of Nottingham, Queen's Medical Centre, Nottingham NG7 2UH, UK

**Keywords:** Survivin, Src, Mitochondria, Cancer

## Abstract

Survivin (also known as BIRC5) is a cancer-associated protein that exists in several locations in the cell. Its cytoplasmic residence in interphase cells is governed by CRM1 (also known as XPO1)-mediated nuclear exportation, and its localisation during mitosis to the centromeres and midzone microtubules is that of a canonical chromosomal passenger protein. In addition to these well-established locations, survivin is also a mitochondrial protein, but how it gets there and its function therein is presently unclear. Here, we show that the first ten amino acids at the N-terminus of survivin are sufficient to target GFP to the mitochondria *in vivo*, and ectopic expression of this decapeptide decreases cell adhesion and accelerates proliferation. The data support a signalling mechanism in which this decapeptide regulates the tyrosine kinase Src, leading to reduced focal adhesion plaques and disruption of F-actin organisation. This strongly suggests that the N-terminus of survivin is a mitochondrial-targeting sequence that regulates Src, and that survivin acts in concert with Src to promote tumorigenesis.

## Abstract (precis)

**Summary:** The proline-rich N-terminal ten amino acids of survivin is a mitochondrial-targeting domain and a regulator of the proto-oncogene Src. Hence, survivin could act in concert with Src to promote tumorigenesis.

## INTRODUCTION

Survivin (also known as BIRC5) is a cancer-associated protein that inhibits cell death and is essential for mitosis (Altieri, 2008). Although its expression is usually confined to G2-phase and mitosis, survivin is often expressed throughout the cell cycle in cancer. Its abundance in tumours correlates with increased resistance to chemotherapy and radiation, treatments lethal to cells through DNA damage and apoptosis induction. When present in interphase, survivin is predominantly cytoplasmic and is actively shuttled out of the nucleus by CRM1 (also known as exportin, XPO1) (Colnaghi et al., 2006; Knauer et al., 2007; Rodríguez et al., 2002; Stauber et al., 2007). Nuclear expression of survivin has been correlated with relapse-free prognosis for some cancer patients (Knauer et al., 2007) and longer survival in others (Okada et al., 2001; Tonini et al., 2005), although several papers argue the opposite (see Wheatley, 2011). Supporting the cytoprotective role of cytoplasmic survivin, we and others have shown that mutating its nuclear export signal, or forcing nuclear location, abrogates cytoprotection from irradiation and apoptosis (Colnaghi et al., 2006; Connell et al., 2008; Knauer et al., 2007), and might have therapeutic potential (Rexhepaj et al., 2010).

In addition to the cytoplasmic and nuclear pools, in cancer cells, some survivin resides in the mitochondria (Dohi et al., 2004). As for other mitochondrial and cytoplasmic proteins (Itoh et al., 2005), when overexpressed the mitochondrial pool of survivin is eclipsed by the abundant cytoplasmic population. However, subcellular fractionation has clearly shown its presence in this organelle, and its abundance increases in response to hypoxia and treatment with adriamycin or etoposide (Ceballos-Cancino et al., 2007; Dohi et al., 2004). Despite its early detection in mitochondria, how survivin enters mitochondria and functions therein remains unclear. Kang et al. (2011) have shown that a cofactor called aryl hydrocarbon (AH)-receptor-interacting protein facilitates entry of survivin into mitochondria by interacting with its C-terminal residue, D142 (Kang et al., 2011). Alternatively survivin might be chaperoned into mitochondria by Hsp90 family proteins, which interact with its baculovirus-inhibitor-of-apoptosis repeat domain (Fortugno et al., 2003).

As mitochondria are instrumental in apoptosis, one might expect the primary function of mitochondrial survivin to relate to its status as an inhibitor of apoptosis protein. In fact, Dohi et al. (2004) have found survivin had to be released from mitochondria to effectively counter cell death. The functional relevance of mitochondrial survivin might also be linked to its interaction with Hsp90 family proteins (Fortugno et al., 2003), as treatment with the survivin–Hsp90 antagonist shepherdin compromises mitochondrial integrity (Hoel et al., 2012; Vishal et al., 2011). Survivin might also influence mitochondrial dynamics by modulating the sculpting proteins, Drp1 (also known as DNM1L) and Fis1 (Hagenbuchner et al., 2013). Either way, one would expect that compromising mitochondrial integrity would affect apoptosis and metabolism (Hagenbuchner et al., 2013; Rivadeneira et al., 2015).

Src is a non-receptor tyrosine kinase that is targeted to the plasma membrane by myristoylation and is frequently overexpressed or aberrantly activated in cancer, particularly epithelial cancers (Frame, 2002; Giaccone and Zucali, 2008). Src, the first proto-oncogene identified, was discovered as the endogenous homologue of the oncogene, v-Src. Src is involved in many cellular events and, like survivin, interfaces life and death at several levels. At the plasma membrane, Src regulates cell–matrix attachment through focal adhesions and the F-actin cytoskeleton. However, somewhat paradoxically, prolonged Src activity prevents focal adhesion turnover causing increased adhesion. Src can be directed to mitochondria by proline-rich cofactors that interact with its SH3 domain including Dok4 (Itoh et al., 2005), and T-cell leukemia virus type-1 protein (Tibaldi et al., 2011).

This study aimed to determine how survivin enters the mitochondria and its function therein. We report that expression of an N-terminal survivin truncation lacking the first ten residues causes increases in the numbers of focal adhesions and abundance of F-actin in cells, which we attribute to its ability to activate Src. Conversely, adhesion is decreased following the expression of the N-terminal decapeptide alone. Finally, we show that the N-terminus is a mitochondrial-targeting sequence (MTS) that binds Src. Collectively, these data suggest that survivin liaises with Src to promote tumorigenesis.

## RESULTS AND DISCUSSION

### Cells expressing survivin_11–142_–GFP are highly adherent

**Fig. 1.**
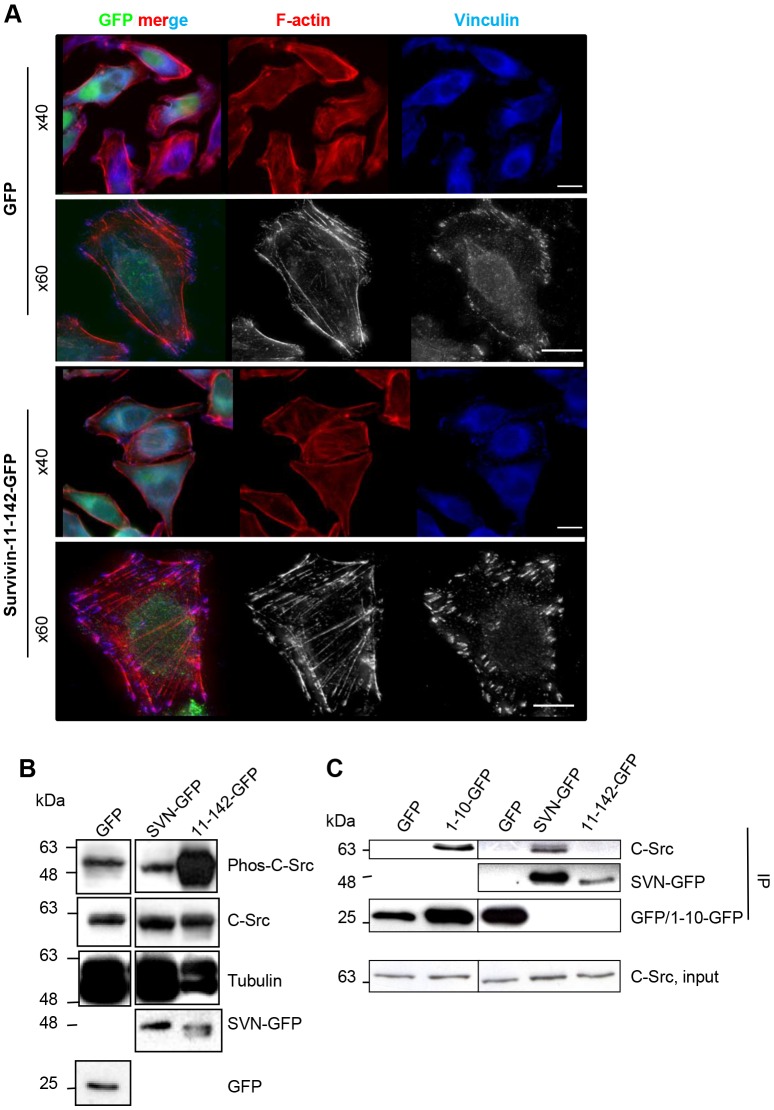
**The N-terminus of survivin regulates Src.** (A) HeLa cells expressing GFP or survivin_11–142_–GFP  (green) were grown on glass coverslips, fixed and probed to visualise focal adhesions with anti-vinculin antibodies (blue) and F-actin with Rhodamine–phalloidin (red). Scale bars: 5 µm. (B) Asynchronous cell lysates immunoblotted with anti-phospho-Src, anti-Src and anti-tubulin antibodies. Anti-GFP verified expression of each construct. (C) Src co-immunoprecipitated (in experiments using GFP Trap beads) with survivin_1–10_–GFP and survivin–GFP, but not GFP or survivin_11–142_–GFP. 11-142-GFP, survivin_11–142_–GFP; 1-10-GFP, survivin_1–10_–GFP;SVN-GFP, survivin–GFP.

We recently showed that HeLa cells expressing an N-terminal truncation of survivin comprising amino acids 11–142, survivin_11–142_–GFP were resistant to apoptosis and sensitised to irradiation (Wheatley, 2015). During handling, we also noticed that they were more adherent than controls, suggesting that focal adhesions were affected. Therefore, we grew cells on glass coverslips, fixed and probed them with anti-vinculin antibodies and counterstained with Rhodamine–phalloidin to visualise F-actin. Compared with GFP controls, survivin_11–142_–GFP cells had more prominent focal adhesions and much stronger F-actin fibres (Fig. 1A).

### The N-terminus of survivin regulates Src activity

It is well established that the formation and dynamics of focal adhesions and F-actin integrity are dependent on Src activity (Frame, 2002). Thus, we examined whether Src activity was altered in these cells. Lysates from cells expressing GFP, survivin–GFP or survivin_11-142_–GFP were assessed for changes in Src expression and activity by immunoblotting with pan-Src antibodies and antibodies against its tyrosine-phosphorylated form (phospho-SrcY416), respectively. Strikingly, although Src was present at similar levels in all samples, its activity was highly elevated in cells expressing survivin_11–142_–GFP (Fig. 1B).

As truncating the first ten residues of survivin had such a profound effect on adhesion and Src activity, we turned our attention to the N-terminus itself. Interestingly, three of these ten residues (residues 4, 6 and 7) are prolines: MGAPPTLPAW. Although enrichment of prolines within this decapeptide might explain why structural data was not forthcoming (Verdecia et al., 2000; Sun et al., 2005), from a functional perspective it suggests the potential to interact with SH3-domain-containing proteins such as Src. To test this, we checked whether these first ten amino acids (survivin_1–10_–GFP), and survivin–GFP, could immunoprecipitate endogenous Src, and gained a positive result in each case (Fig. 1C). Mediation of Src–survivin interaction by the N-terminus was further corroborated by the inability of survivin_11–142_–GFP, to co-immunoprecipitate Src in this experiment. Thus, we conclude that these N-terminal ten residues are necessary and sufficient to bind to Src.

### Survivin_1–10_ is a MTS

**Fig. 2.**
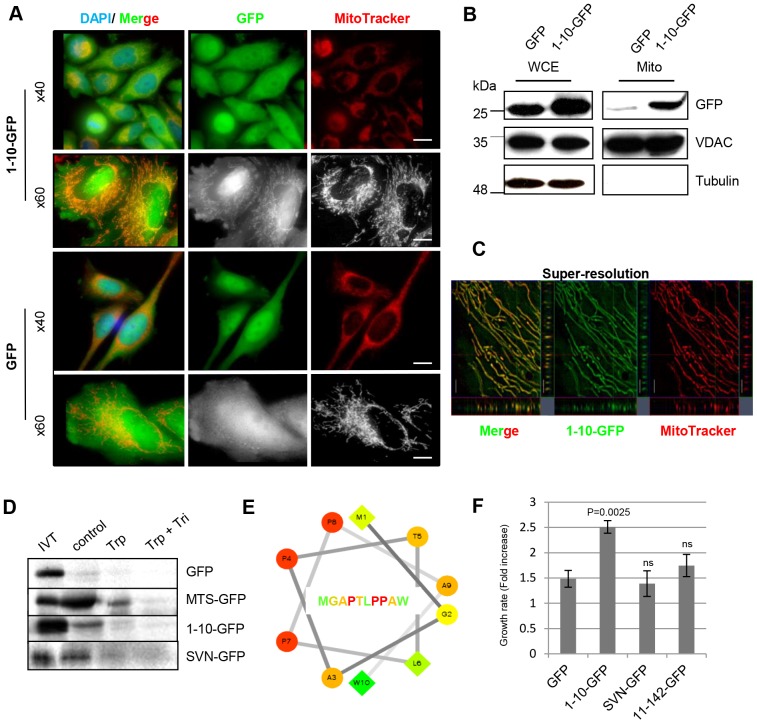
**The first ten amino acids of survivin are an MTS.** (A) HeLa cells expressing survivin_1–10_–GFP (1-10-GFP) or GFP (green) were grown on poly-L-lysine-coated slides, stained with MitoTracker (red) and imaged live. Scale bars: 20 µm (upper) and 5 µm (lower). (B) Immunoblot of fractionated cells: 25 µg each of whole-cell extracts (WCE) and the cytoplasmic fraction (cyto), and 8 µg of the mitochondrial fraction (mito). Anti-GFP antibody detects GFP and survivin_1–10_–GFP, and anti-VDAC and anti-tubulin antibodies highlight mitochondrial and cytoplasmic fractions, respectively. (C) Structured illumination of mitochondria in live cells expressing survivin_1–10_–GFP stained with MitoTracker. Scale bar: 1 µm. (D) Mitochondrial import assay: GFP, MTS–GFP, survivin_1–10_–GFP and survivin–GFP translated *in vitro* (IVT), labelled with [^35^S]methionine then incubated with mitochondria isolated from HeLa cells for 1 h at 37°C. Mitochondria were washed in isolation buffer (control) or treated with 150 µg/ml trypsin (trp) or trypsin and 1% Triton X-100 (tri). Mitochondrial retention of proteins was assessed by SDS-PAGE and phosphor-imaging. (E) Hydropathy wheel plot of the first ten residues of survivin. Residue type: circles, hydrophilic; diamonds, hydrophobic. Hydrophobicity scale is green (high) to yellow (zero). Hydrophilicity scale is red (high) to orange (low). (F) The rate of cell growth of each line was compared in exponential phase. Mean±s.d. of three independent experiments is shown. A paired *t*-test demonstrated that the increased growth rate of 1-10-GFP cells is significantly different from the GFP control, variance in other lines was not significant (ns). 11-42-GFP, survivin_11–142_–GFP; SVN-GFP, survivin–GFP.

By fusing the first 30 nucleotides of the human survivin gene to GFP cDNA we engineered MGAPTLPPAW–GFP. When expressed in HeLa cells we discovered that it localised to mitochondria (Fig. 2A). To verify this biochemically, cells expressing GFP or survivin_1–10_–GFP were fractionated by differential centrifugation, and whole-cell extracts and mitochondrial-enriched fractions were probed with anti-GFP antibodies (Fig. 2B). Anti-VDAC and anti-tubulin antibodies were used to identify mitochondrial fractions and cytoplasmic contamination, respectively. Survivin_1–10_–GFP was clearly present in the mitochondrial fraction, whereas GFP was excluded (Fig. 2B). Next, super-resolution microscopy was used to image survivin_1–10_–GFP in living cells. Structured illumination showed that survivin_1–10_–GFP was coincident with MitoTracker throughout the mitochondria rather than simply binding to the exterior (Fig. 2C). To ascertain whether survivin_1–10_–GFP could be targeted to the mitochondria independently of any cofactors, we *in vitro* translated (IVT) [^35^S]methionine-labelled GFP or survivin_1–10_–GFP to compare their import into isolated mitochondria, using the MTS of cytochrome*c* oxidase subunit VIIIA as a positive control (MTS–GFP). Fig. 2D shows the IVT protein and radiolabelling, followed by assessment of association of each protein with the mitochondria after washing in buffer (control), after incubation in trypsin (to remove exteriorly bound proteins), or after trypsin and Triton X-100, which eliminates all proteins, GFP acted as negative control; its signal was low after the control wash, and eliminated by trypsin treatment, highlighting its failure to be imported. In contrast, MTS–GFP, survivin_1–10_–GFP and survivin–GFP were successfully imported into the mitochondria, as evidenced by protein remaining after trypsin treatment. These data suggest that survivin_1–10_–GFP and survivin–GFP are mitochondrial residents and can enter this organelle independently of cofactors.

MTSs are normally N-terminally placed amphiphilic stretches of 17–40 amino acids that tend to form amphipathic α-helices that engage with translocase complexes of the outer and inner mitochondrial membrane. The mitochondrial localisation of survivin_1–10_ and its ability to access isolated mitochondria *in vitro* suggest that it is a bona fide MTS despite its short length. Consistent with this, survivin_1–10_ conforms to the amphiphilic requirements of a canonical MTS when mapped on a hydropathy plot (Fig. 2E), with hydrophobic residues predominantly on one side, and hydrophilic residues on the other.

### The N-terminus of survivin regulates substrate adhesion

**Fig. 3.**
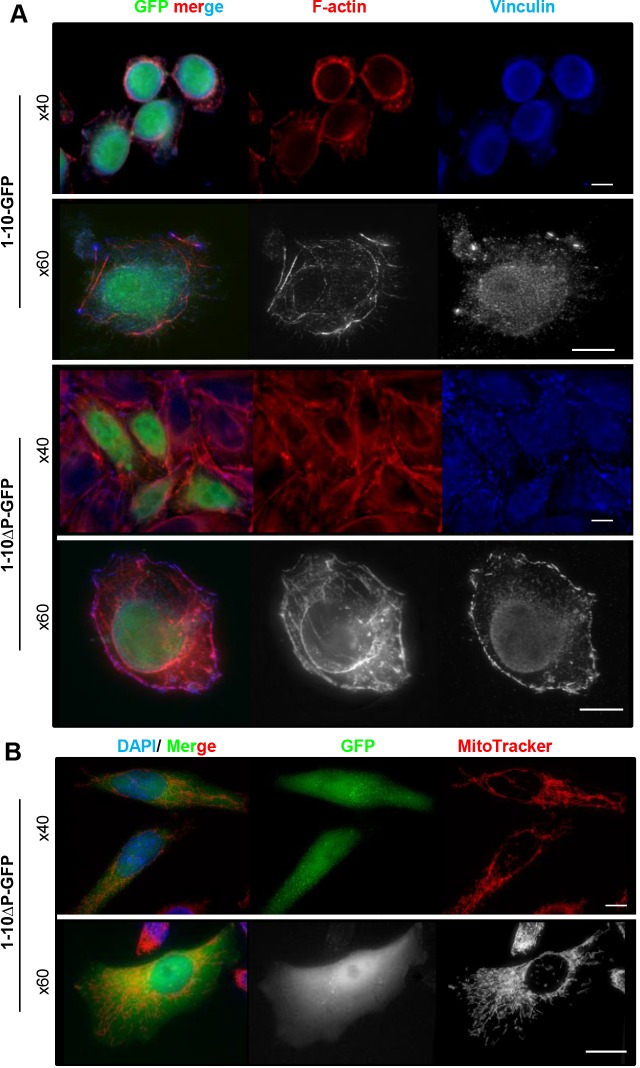
**Survivin_1-10_–GFP is a proline-rich sequence that reduces cell adhesion.** (A) Cells expressing survivin_1-10_ –GFP or survivin_1-10ΔP_-GFP (green, 1-10-GFP and 1-10ΔP-GFP, respectively) were grown on uncoated glass coverslips, stained as in Fig. 1A and viewed with objectives of the indicated magnification. (B) Live cells expressing survivin_1-10ΔP_–GFP stained with MitoTracker viewed with objectives of the indicated magnification. Scale bars: 10 µm.

While handling, we noted that cells expressing survivin_1–10_–GFP grew more rapidly than controls (Fig. 2F) and were less adherent (Fig. 3A). To determine whether the proline residues were required for adhesion, we replaced them with alanine residues (survivin_1–10ΔP_–GFP) and transiently expressed this mutated protein into HeLa cells. Proline-to-alanine replacement restored focal adhesions and F-actin assembly (Fig. 3A). Moreover, when observed live, the percentage of unspread or floating cells was reduced from 96.2% (*n*=104) cells expressing survivin_1–10_–GFP, to 10.8% (*n*=277) in cells expressing survivin_1–10ΔP_–GFP. Live imaging also revealed that mitochondrial targeting was abolished by proline-to-alanine mutation (Fig. 3B). The presence of both a proline-rich sequence and a MTS in the N-terminus fits with a precedent described for Dok4 (Itoh et al., 2005) and HTLV1 (Tibaldi et al., 2011), suggesting that it is a Src-regulator and a mitochondrial chaperone.

### Conclusion

We report the novel findings that the N-terminus of survivin is both a Src regulator and an MTS. The data suggest that much of what survivin achieves in cancer might be accomplished in collaboration with Src.

## MATERIALS AND METHODS

Unless otherwise indicated, tissue culture reagents were obtained from Invitrogen, cloning enzymes from NEB, and all other reagents from Sigma-Aldrich.

### Molecular cloning

Wild-type survivin_1–10_–GFP was generated by annealing two primers corresponding to the first 30 nucleotides of human survivin cDNA, with 5′ EcoRI and 3′ HindIII sites. The annealed DNA fragment was ligated into pBS-GFP then shuttled into pcDNA3.1 (Invitrogen) using EcoRI and Xho1. The triple mutation that translates to MGAaTLaaAW (lowercase letters indicate the mutated residues) was made by site-directed mutagenesis with the 5′ primer 5′-ATGGGTGCCGCGACGTTGGCCGCTGCCTGG-3′ and 3′ primer 5′-CCAGGCAGCGGCCAACGTCGCGGCACCCAT-3′ (Eurofins, MWG Operon), Vent polymerase, dNTPs and survivin_1–10_–GFP cDNA as template, using Stratagene Quickchange II kit (Agilent Technologies). The template was digested with DpnI and nascent cDNA transformed into competent DH5α *E.coli* cells. All sequences were verified prior to use.

### Cell culture and proliferation

HeLa cells (derived from ATCC stock) were cultured at 37°C in 5% CO_2_ humidified incubator in Dulbecco's Modified Eagle's Medium (DMEM) with 10% HyClone fetal bovine serum (FBS), L-glutamine (2 mM), 1% penicillin-streptomycin and 1% fungizone. To create lines stably expressing GFP-tagged proteins, cells in antibiotic-free DMEM were transfected with pcDNA3.1 constructs using FuGENE 6 (Promega) in Opti-MEM. To select for positive transformants, G418 (50 μg/ml) was added 24 h post transfection and sorted by FACS. Cell number was assessed using a resazurin-based assay in which cells were incubated for 1 h at 37°C in 10 μg/ml resazurin in DMEM and measured spectrophotometrically (FLUOstar Galaxy, BMG Labtechnologies) with excitation at 530 nm and emission at 590 nm.

### Mitochondrial assays

#### Fractionation

10^6^ cells were resuspended in mitochondrial isolation buffer (10 mM HEPES, pH 7.5, 200 mM mannitol, 1 mM EGTA and 70 nM sucrose with protease inhibitors), and lysed with 25 strokes in a 2-cm^3^ glass homogeniser. Nuclei were removed by a 5-min centrifugation at 1000 ***g***. The supernatant was re-spun (2000 ***g***) to remove contaminating nuclei, then spun at 10,000 ***g***, (15 min, 4°C) to pellet mitochondria, which were re-washed and pelleted two more times to ensure purity.

#### Import

GFP, MTS–GFP, survivin_1–10_–GFP and survivin–GFP were translated *in vitro* (IVT) from pcDNA templates using T7 RNA polymerase, incorporating [^35^S]methionine using a rabbit reticulocyte lysate system (Promega). Radiolabelled proteins were incubated for 1 h at 37°C with mitochondria isolated from HeLa cells in import buffer [20 mM HEPES pH7.5, 3% (w/v) fatty acid-free BSA, 250 mM sucrose, 80 mM KCl, 5 mM MgCl_2_ supplemented with 2 mM ATP and 10 mM sodium succinate], before washing in buffer or incubation in 150 µg/ml trypsin or trypsin plus 1% Triton X-100 (15 min, on ice).

### Immunoblotting

Cell lysates were prepared in M-PER (ThermoFisher, 45 min, room temperature), with standard protease (1 µg/ml) and phosphatase inhibitors including 2 mM sodium orthovanadate. Standard procedures were used for SDS-PAGE (12%) and transfer to nitrocellulose (PALL). To detect GFP-tagged proteins, membranes were probed with anti-GFP antibodies (catalogue no. 11814460001, 1:1000, Roche). Additional primary antibodies used were against: tubulin (B512, 1:2000, Sigma); Src (SC-18, 1:1000, Santa Cruz Biotechnology); phospho-SrcY416 (catalogue no. 6943, 1:1000, Cell Signalling); VDAC (D73D12, 1:1000, Cell Signalling). Incubations were carried out in PBS with 5% milk and 0.1% Tween 20, except for phospho-SrcY416, for which TBST and 5% BSA was used. Horseradish peroxidise (HRP)-conjugated secondary antibodies (DAKO, 1:2000), enhanced chemiluminescence (GeneFlow) and X-ray film (GE Healthcare) were used to detect bands.

### Immunoprecipitation

Cells (3×10^6^) were harvested by scraping and lysed in 200 µl lysis buffer (10 mM Tris-HCl pH 7.5, 150 mM NaCl, 0.5 mM EDTA, 0.5% NP-40) supplemented with standard protease inhibitors, 2 U benzonase and 2 mM MgCl_2_. Lysates were clarified by centrifugation (20,000 ***g***, 2 min, 4°C) then diluted in dilution buffer (10 mM Tris-HCl pH 7.5, 150 mM NaCl, 0.5 mM EDTA). For every 500 µl of extract, 25 µl of prewashed GFP-trap_A beads (50% slurry, Chromotek) were added (note that the exact quantity was optimised to the expression of GFP-tagged protein in each sample). Lysates and beads were incubated for 1 h at 4°C with rotation, then pelleted by centrifugation at 2500 ***g*** for 2 min at 4°C and washed in ice-cold dilution buffer. Proteins were boiled off the beads (95°C for 10 min) in lysis buffer with SDS-sample buffer.

### Microscopy

#### Fixed-cell imaging

Cells were cultured on glass coverslips with or without poly-L-lysine, then fixed with 4% formaldehyde, permeabilised using 0.15% Triton X-100 in PBS, and blocked with 1% BSA before immunoprobing with anti-vinculin antibodies (catalogue no. 73614, 1:1000, Santa Cruz Biotechnology, 1 h room temperature), and Cy5-secondary anti-rabbit-IgG antibodies (1:1000, AbCam; 1 h room temperature). Samples were counterstained with 20 nM Rhodamine–phalloidin and DAPI, then mounted with Mowiol. Images were acquired using an inverted (Olympus IX71) microscope with 40× NA1.2 oil and 60× NA1.4 oil objectives, DeltaVision software (GE Healthcare) and a Coolsnap HQ^2^ camera (Photometrics). Maximum projections of deconvolved 0.3-μm *z*-stacks prepared in Photoshop are presented.

### Live-cell imaging

Cells were grown in glass-bottomed dishes (Willco) with or without poly-L-lysine. Prior to imaging, medium was replaced with MitoTracker^®^ CMXRos (25 nM) in Phenol-Red-free CO_2_-independent medium and imaged as above. For super-resolution microscopy, a Zeiss Elyra PS.1 microscope was used in structured illumination mode, with the following settings: objective Plan-Apochromat 63×1.4 oil DIC M27, filter set LBF-488/561 and cmos camera exposure time 20 ms. Two imaging tracks were set up in fast frame mode, which alternates the excitation lasers (solid state 488 nm and 561 nm at 20% and 10% laser power settings, respectively). Channel alignment was confirmed using 100-nm beads scanned with the same settings. Image processing and alignment was carried out using Zeiss Zen Black 2012 software.
